# Supplementary material for: Accelerating Multiparametric Quantitative MRI Using Self‐Supervised Scan‐Specific Implicit Neural Representation With Model Reinforcement
Source: Magn Reson Med. 2025 Dec 19;95(5):2963–79. doi: 10.1002/mrm.70227 (PMC12962221; doi:10.1002/mrm.70227)
Supplement: Supplementary file 1 — Data S1. An Overview of Hash Encoding. Figure S1. Illustration of the model adaptation strategy. (A) For the initialization module, we reuse and freeze the temporal features and MLP weights from previously reconstructed data. (B) For the physics reinforcement module, we incorporate a low‐rank adaptation (LoRA) strategy that freezes most parameters in U‐Net and only learns a small set of parameters. Figure S2. Undersampling masks for in vivo and phantom experiments. (A) 2D variable density Gaussian undersampling patterns with AF = 4 and AF = 5 for in vivo experiments. (B) A 1D variable density Gaussian undersampling pattern with AF = 4 for phantom experiments. Figure S3. Bland–Altman plots for (A) RELAX and (B) MANTIS across five ROIs. The solid lines indicate mean differences, and the dashed lines represent the 95% confidence level. Table S1. Reconstruction times of different methods evaluated on the in vivo dataset. The values indicate the average runtime required for a single data reconstruction. The annotations in parentheses specify the computational device used for each method (CPU or GPU). The proposed REFINE‐MORE shows a substantially reduced runtime after applying the model adaptation strategy. Figure S4. Reconstruction results at AF = 4 using zero‐filling initialization and the proposed REFINE‐MORE with INR initialization. Quantitative metrics (nRMSE and SSIM) on this subject are reported below each reconstruction. INR‐initialized REFINE‐MORE generates parameter maps with reduced artifacts and closer agreement with the fully sampled reference. [file MRM-95-2963-s001.docx]

**Supporting Background Information.** An Overview of Hash Encoding.

**Hash encoding with multilayer perceptron as the representation function**

In this study, we employ a hash encoding^1^ and an MLP as the continuous function to represent the images to be solved. Hash encoding maps the low-dimensional coordinates into a high-dimensional feature space, thereby facilitating the learning of high-frequency details. Compared to other encoding strategies in INR, hash encoding incorporates learnable parameters that permit the use of a much smaller MLP architecture while maintaining high representational capacity. This leads to faster convergence during training and improves memory efficiency, making it feasible to model and process entire 4D volumes on standard GPU hardware. Specifically, the learnable parameters in hash encoding are structured across $L$ distinct resolution levels, each implemented as a separate hash table. These levels span a range of geometrically increasing resolutions, starting from a base resolution $N_{min}$ and scaling by a factor of $b$ at each successive level. As a result, the resolutions follow the sequence $N_{min}$, $b\times N_{min}$, …, $b^{L-1}\times N_{min}$. Each resolution level is composed of $T$ feature entries, and the dimensionality of each feature vector is $F$. Therefore, hash encoding provides a multi-resolution representation that enables hierarchical feature learning. Benefitting from the fast convergence of hash encoding combined with MLPs, INR can rapidly provide reasonable initialization estimates of 3D quantitative maps. The hyperparameters in hash encoding were fixed as the default values. For a more detailed explanation of hash encoding, readers may refer to the original paper.


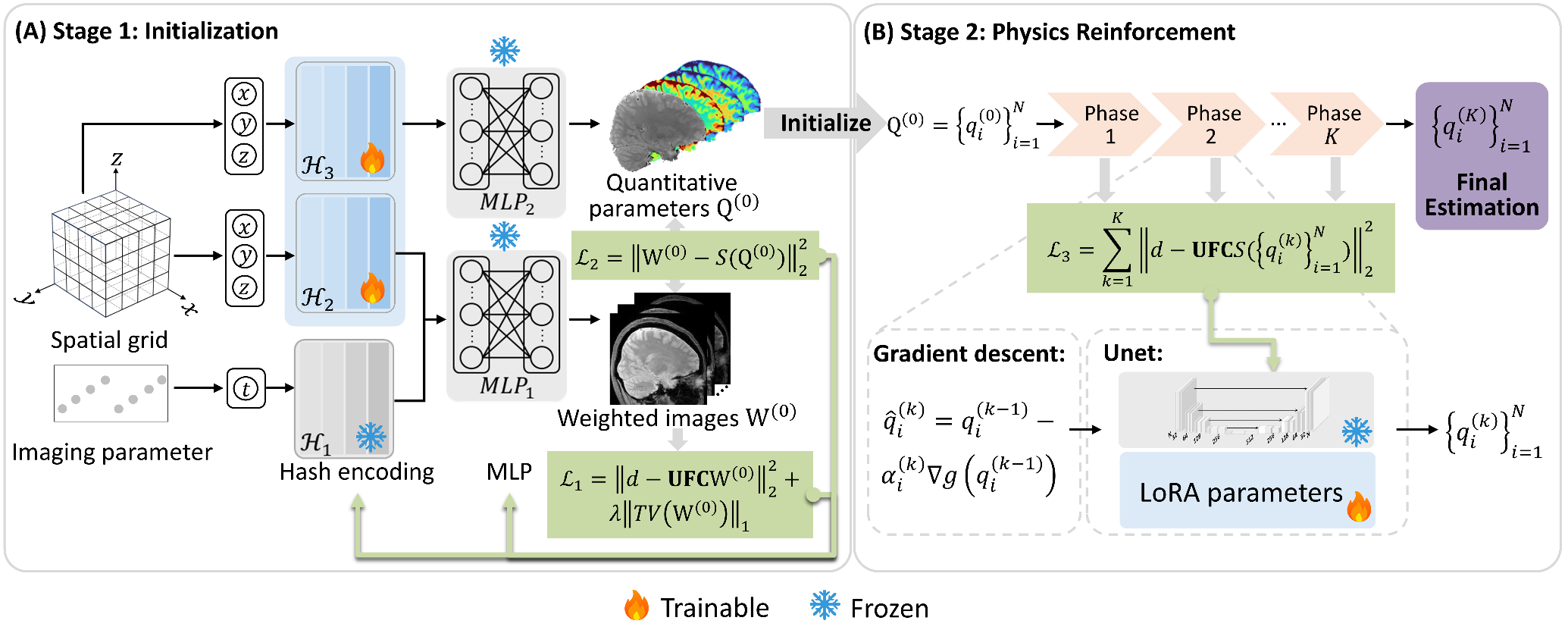


**Supporting Information Figure S1.** Illustration of the model adaptation strategy. (A) For the initialization module, we reuse and freeze the temporal features and MLP weights from previously reconstructed data. (B) For the physics reinforcement module, we incorporate a low-rank adaptation (LoRA) strategy that freezes most parameters in U-Net and only learns a small set of parameters.


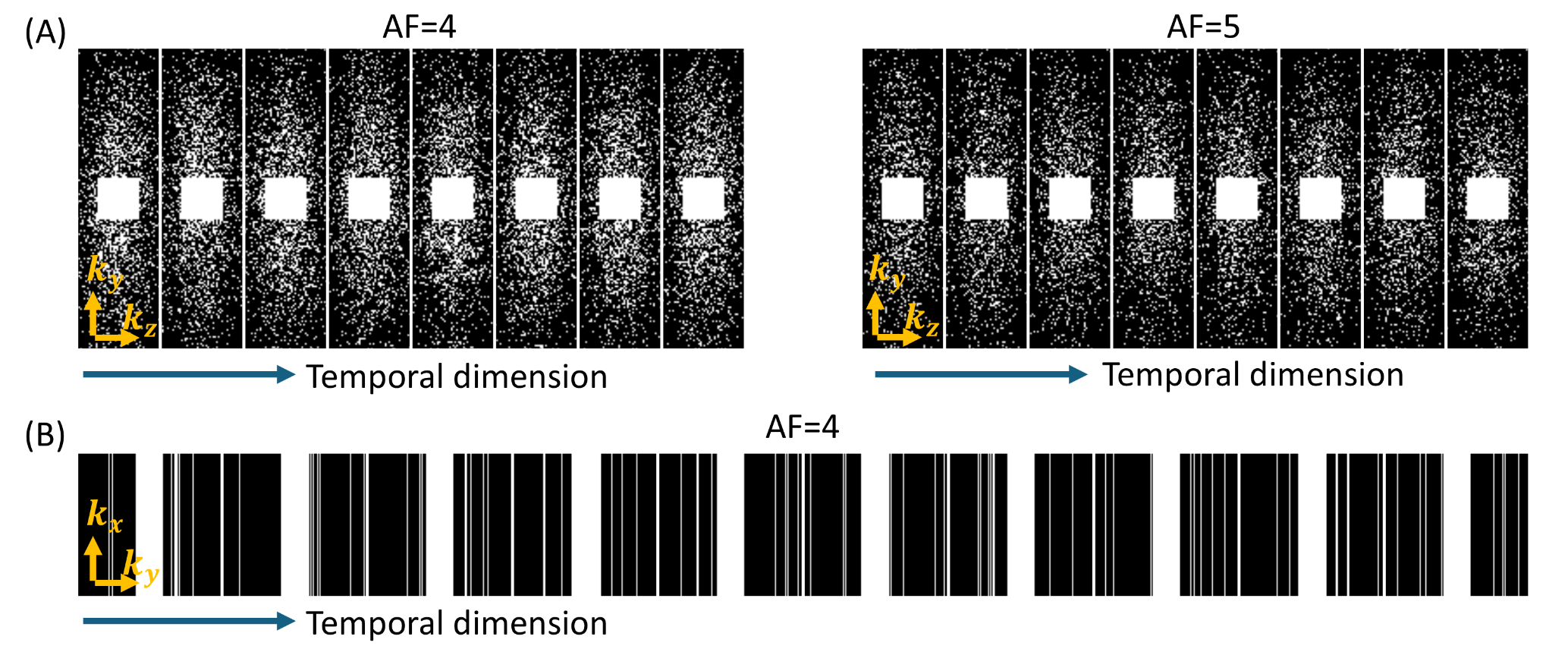


**Supporting Information Figure S2.** Undersampling masks for in vivo and phantom experiments. (A) 2D variable density Gaussian undersampling patterns with AF=4 and AF=5 for in vivo experiments. (B) A 1D variable density Gaussian undersampling pattern with AF=4 for phantom experiments.


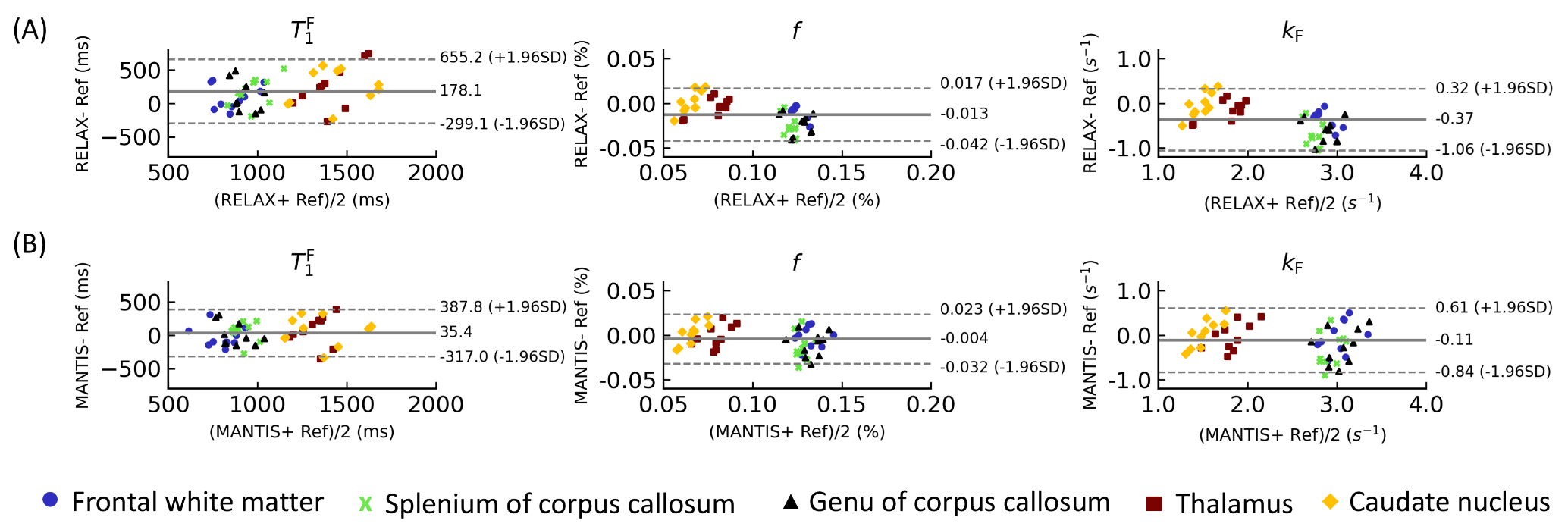


**Supporting Information Figure S3.** Bland-Altman plots for (A) RELAX and (B) MANTIS across five ROIs. The solid lines indicate mean differences, and the dashed lines represent the 95% confidence level.

**Supporting Information Table S1.** Reconstruction times of different methods evaluated on the in vivo dataset. The values indicate the average runtime required for a single data reconstruction. The annotations in parentheses specify the computational device used for each method (CPU or GPU). The proposed REFINE-MORE shows a substantially reduced runtime after applying the model adaptation strategy.

| Zero Filling (CPU) | 4.1 min |
| --- | --- |
| LLR (CPU) | 6.2 min |
| RELAX (GPU) | 14.9 min (Training), 0.04 s (Testing) |
| MANTIS (GPU) | 2.5 min (Training), 0.03 s (Testing) |
| SUMMIT (GPU) | 11.5 min |
| REFINE-MORE (GPU) | 71.8 min (Without adaptation), 14.2 min (With adaptation) |


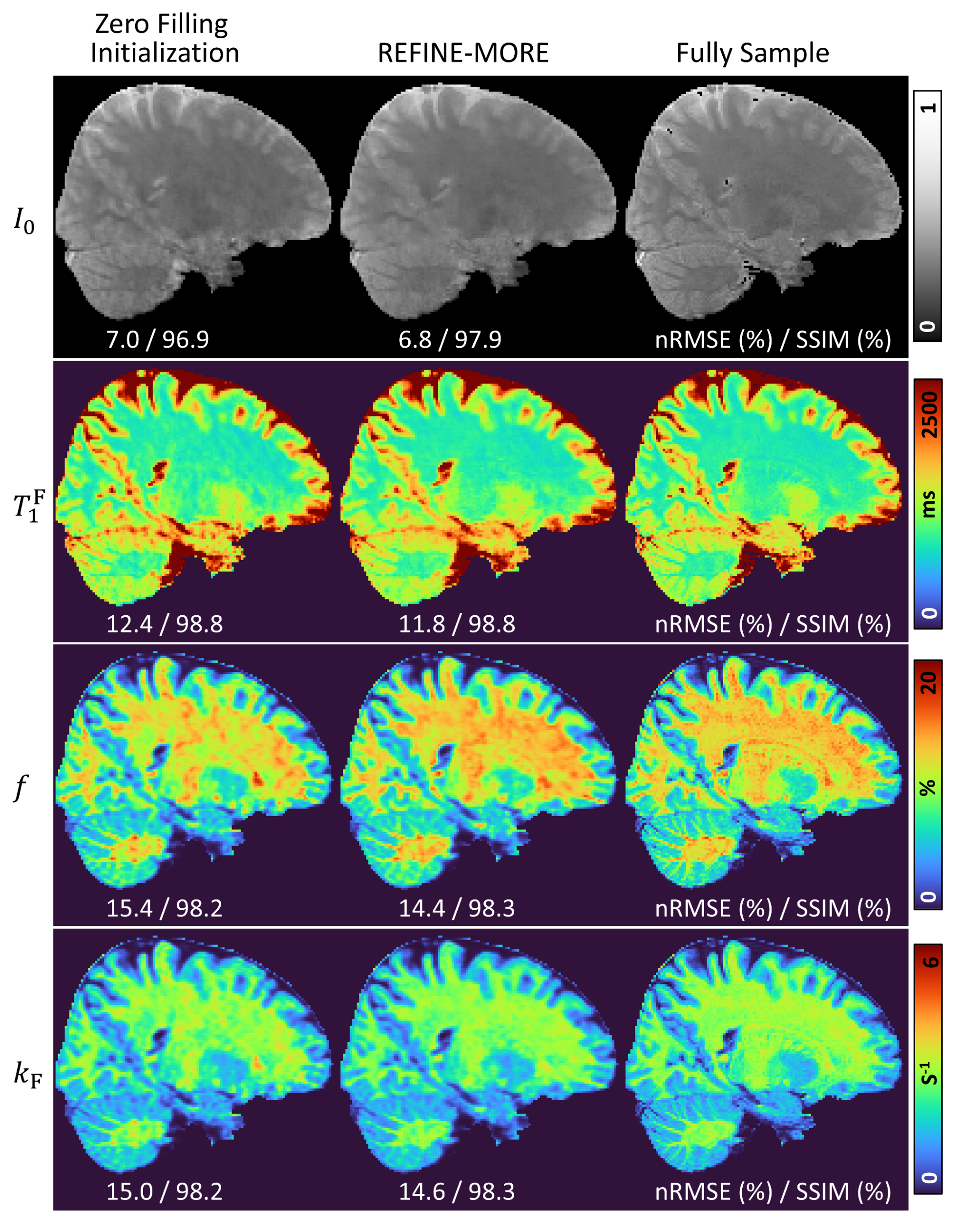


**Supporting Information Figure S4.** Reconstruction results at AF=4 using zero-filling initialization and the proposed REFINE-MORE with INR initialization. Quantitative metrics (nRMSE and SSIM) on this subject are reported below each reconstruction. INR-initialized REFINE-MORE generates parameter maps with reduced artifacts and closer agreement with the fully sampled reference.

**Reference**

1. Müller T, Evans A, Schied C, Keller A. Instant Neural Graphics Primitives with a Multiresolution Hash Encoding. ACM Trans Graph. 2022;41(4):1-15. doi:10.1145/3528223.3530127
